# Roles of the PI-3K and MEK pathways in Ras-mediated chemoresistance in breast cancer cells

**DOI:** 10.1038/sj.bjc.6601048

**Published:** 2003-07-01

**Authors:** W Jin, L Wu, K Liang, B Liu, Y Lu, Z Fan

**Affiliations:** 1 Department of Experimental Therapeutics, The University of Texas MD Anderson Cancer Center, Houston, TX 77030, USA

**Keywords:** breast cancer, drug resistance, Ras, Akt, MAPK

## Abstract

Activated Ras utilises several downstream pathways, including the mitogen-activated protein kinase (MAPK) kinase (MEK)/MAPK pathway and the phosphoinositide 3-kinase (PI-3k)/Akt pathway, to promote cell proliferation and to inhibit apoptosis. To investigate which pathway plays a major role in Ras-induced drug resistance to chemotherapeutic agents in breast cancer cells, we transfected MCF7 breast cancer cells with a constitutively active H-RasG12V and examined the toxicities of three commonly used breast cancer chemotherapeutic agents, paclitaxel, doxorubicin, and 5-fluorouracil in these cells under the conditions that PI-3K or MEK were selectively inhibited by their respective specific inhibitors or dominant negative expression vectors. We found that Ras-mediated drug resistance is well correlated with resistance to apoptosis induced by anticancer agents in MCF7 breast cancer cells. Although inhibition of MEK/MAPK or PI-3K/Akt can each enhance the cytotoxicity of paclitaxel, doxorubicin, or 5-fluorouracil, inhibition of the PI-3K/Akt pathway seems to have a greater effect than inhibition of the MEK/MAPK pathway in reversing Ras-mediated drug resistance. Our results indicate that the PI-3K pathway may play a more important role in receptor tyrosine kinase-mediated resistance to chemotherapy and suggest that PI-3K/Akt might be a critical target molecule for anticancer intervention in breast cancer.

It has been well-demonstrated that chemotherapy is effective in prolonging both disease-free and overall survival of women with breast cancer. A further question is how to improve the existing regimens of breast cancer chemotherapy for better therapeutic outcomes. Recent studies have provided convincing evidence that the therapeutic outcome of chemotherapy may be affected by the expression and activity of receptor tyrosine kinases such as epidermal growth factor (EGF) receptor, HER2, and their related molecules. Pursuance of this concept may provide important clues for optimising the clinical applications of breast cancer chemotherapy, and for designing new therapeutic approaches.

Members of the Ras superfamily of GTPases act as important molecular switches to coordinate extracellular stimuli with activation of intracellular signalling pathways for appropriate biological responses. Although Ras genetic mutational activation is infrequent in breast cancer, Ras is often pathologically activated in breast cancer due to its link to the EGF receptor family or other tyrosine kinases that signal through Ras and that are commonly overexpressed in this disease (Kroll *et al*, 2002). Activated Ras status has been associated with reduced oestrogen dependence and increased invasiveness of breast cancer (Kasid *et al*, 1985; Sukumar *et al*, 1988; Sommers *et al*, 1990). Owing to this important role of Ras in tumorigenesis, the Ras-signalling pathway has attracted considerable attention as a target for anticancer therapy. Novel cancer therapeutic approaches based on the inhibition of Ras-mediated signalling, including inhibition of Ras processing, inhibition of Ras protein synthesis, and blockage of downstream Ras effectors, are being evaluated (Adjei, 2001).

Activated Ras is involved not only in tumour progression but also possibly in the development of resistance of tumour cells to chemotherapy and to ionising radiation. While the role of Ras in mediating resistance to radiation has been relatively well-studied and is noncontroversial (Gupta *et al*, 2001), the exact role of Ras in mediating resistance to various chemotherapeutic agents is relatively less documented, and in fact, remains controversial or even paradoxical. Constitutive expression of the H-Ras oncogene inhibits doxorubicin-induced apoptosis in a rat rhabdomyosarcoma cell line (Nooter *et al*, 1995). In contrast, the expression of RasG12V in FR3T3 rat fibroblasts sensitised the cells to treatment with cisplatin (Viktorsson *et al*, 2000). Similarly, another recent study found that, in the absence of multidrug resistance, human tumour cell lines with activated Ras oncogenes were uniformly more sensitive to most topoisomerase II inhibitors than were cell lines containing wild-type Ras alleles (Koo *et al*, 1999).

In the setting of human breast cancer, the mechanisms of drug resistance associated with advanced or metastatic hormone-independent breast carcinoma are not yet fully understood. The human breast carcinoma MCF7 cell line represents a typical *in vitro* model of breast cancer with positive oestrogen receptors; the original MCF7 cells are hormone-dependent and noninvasive and become hormone-independent and invasive when these cells are transformed with an activated Ras oncoprotein (Kasid *et al*, 1985; Sukumar *et al*, 1988; Sommers *et al*, 1990). In the present study, we investigated the development of drug resistance in MCF7 cells associated with the expression of an activated Ras (RasG12V) in these cells. As expected, the expression of RasG12V in MCF7 cells improved the survival of MCF7 cells in a hormone-depleted culture medium. We found that, compared with control-vector-transfected cells, the MCF7RasG12V cells exhibited increased resistance to several chemotherapeutic agents currently used for treating breast cancer patients. We further compared the role of two well-known Ras downstream pathways, the PI-3K/Akt pathway and the MAPK/ERK kinase (MEK)/MAPK pathway, in drug resistance mediated by activated Ras. The results of our current study provide further evidence for targeting growth factor receptor and Ras-mediated signal transduction as an approach for enhancing the therapeutic outcome of current breast cancer chemotherapy.

## MATERIALS AND METHODS

### Antibodies and reagents

Antibodies directed against total Akt, ser473-phosphorylated Akt1, and thr202-phosphorylated MAPK (p42/p44) were obtained from Cell Signaling Technology, Inc. (Beverly, MA, USA), anti-Ras antibody was from BD Bioscience Transduction Laboratory (San Jose, CA, USA), anti-MAPK (Erk2) antibody was from Santa Cruz Biotechnology Inc. (Santa Cruz, CA, USA), anti-His G antibody and anti-HA antibody were from Roche Diagnostics Corp (Indianapolis, IN, USA), and LY294002 and PD98059 were obtained from CalBiochem Corp. (San Diego, CA, USA). The antineoplastic agents used in this study were paclitaxel (Taxol) (Bristol Laboratories, Princeton, NJ, USA), doxorubicin (Adriamycin) (Gensia Sicor Pharmaceuticals, Irvine, CA, USA), and 5-fluorouracil (Adrucil) (Pharmacia, Kalamazoo, MI, USA). All other reagents were purchased from Sigma Chemical Co. (St Louis, MO, USA) unless otherwise specified.

### Cells, cell culture, and transfection

MCF7 human breast cancer cells were obtained from American Type Culture Collection (Manassas, VA, USA). The cells were grown and routinely maintained in Dulbecco's modified Eagle's medium/F12 medium supplemented with 10% foetal bovine serum (FBS), 2 mM glutamine, 100 U ml^−1^ penicillin, and 100 *μ*g ml^−1^ streptomycin. Cells were incubated at 37°C with 5% CO_2_ and 95% air.

The RasG12V cDNA (pSR*α*-H-RasV12, which was kindly provided by Dr Richard AJ Janssen), was subcloned into pcDNA3.1 expression vector (Invitrogen, Carlsbad, CA, USA). pcDNA3.1 RasG12V or pcDNA3.1 backbone vector was transfected into MCF7 cells with the Fugene-6 transfection kit (Roche Diagnostics), followed by selection with a medium containing 1000 *μ*g ml^−1^ neomycin (G418), and maintenance in medium containing 400 *μ*g ml^−1^ G418. Stable clones were evaluated for his-tag expression by Western blot analysis with anti-His G antibodies, or anti-Ras antibodies.

Cell proliferation assay was performed by seeding cells onto six-well tissue culture plates at a density of 2 × 10^4^ cells well^−1^, followed by a 5-day culture in medium supplemented with FBS (10 or 0.5%) or charcoal-stripped (CS) FBS (10 or 0.5%). Cells were counted with a Coulter counter (Coulter Corp., Miami, FL, USA).

### Western blot analysis

Cells were lysed in a lysis buffer containing 50 mM Tris, pH 7.4, 150 mM NaCl, 0.5% NP-40, 50 mM NaF, 1 mM Na_3_VO_4_, 1 mM phenylmethylsulphonyl fluoride, 25 *μ*g ml^−1^ leupeptin, and 25 *μ*g ml^−1^ aprotinin. The lysates were cleared by centrifugation, and the supernatants were collected. Equal amounts of lysate protein were used for the Western blot analyses with the indicated antibodies (Fan *et al*, 1995). Specific signals were visualised using the enhanced chemoluminescence (ECL) detection kit (Amersham, Arlington Heights, IL, USA).

### Chemotherapy cytotoxicity assay

Cells were seeded onto 24-well culture plates. After a 4-h pulse exposure of the cells to various doses of chemotherapeutic agents, the cells were cultured for an additional 7 days with drug-free medium containing 10% CS-PBS. Cell viability was then assessed by adding 50 *μ*l of 10 mg ml^−1^ MTT (3-[4,5-dimethylthiazol-2-yl]-2,5-diphenyltetrazolium bromide) (Sigma) to 0.5 ml of culture medium and incubating the cells for 3 h at 37°C in a CO_2_ incubator, followed by cell lysis with 500 *μ*l of lysis buffer containing 20% sodium dodecyl sulphate (SDS) in dimethyl formamide/H_2_O (1 : 1, v v^−1^), pH 4.7, at 37°C for more than 6 h. Cell viability was then determined by measuring the optical absorbance of cell lysates at a wavelength of 595 nm and normalising the value with the value of the corresponding control (untreated cells).

### Quantification of apoptosis by enzyme-linked immunosorbent assay (ELISA)

An apoptosis ELISA kit (Roche Diagnostics) was used to quantitatively measure cytoplasmic histone-associated DNA fragments (mononucleosomes and oligonucleosomes) after induced cell death. This photomeric enzyme immunoassay was performed exactly according to the manufacturer's instructions.

### Terminal deoxynucleotidyl transferase (TdT)-mediated dUTP-X nick end labelling (TUNEL) assay

The TUNEL assay was performed as described previously (Liu *et al*, 2000). Briefly, following fixation with 1% paraformaldehyde on ice for 1 h, the cells were washed once with PBS and then postfixed in ice-cold 70% ethanol overnight. The next day, the cells were washed once with PBS before incubation with 50 *μ*l of TdT reaction solution containing 5 U of TdT, 0.5 nmol of biotin-dUTP, and 2.5 mM CoCl_2_ in TdT buffer (Roche Diagnostics) at 37°C for 1 h. After the reaction, the cells were stained with a buffer containing 2.5 *μ*g ml^−1^ FITC-avidin, 0.1% Triton X-100, 5% dried low fat milk in 4 × SSC (0.6 M sodium chloride-60 mM sodium citrate, pH 7) in the dark at room temperature for 1 h. Before flow cytometric analysis (FCM) with a FACScan flow cytometer (Becton Dickinson, San Jose, CA, USA), the cells were counterstained with a solution containing 5 *μ*g ml^−1^ propidium iodide and 10 *μ*g ml^−1^ RNase A in PBS. The FITC signal was analysed using Epics Elite software (Coulter Corp.).

## RESULTS

### Characterisation of MCF7 breast cancer cells expressing an activated Ras

**Figure 1:**
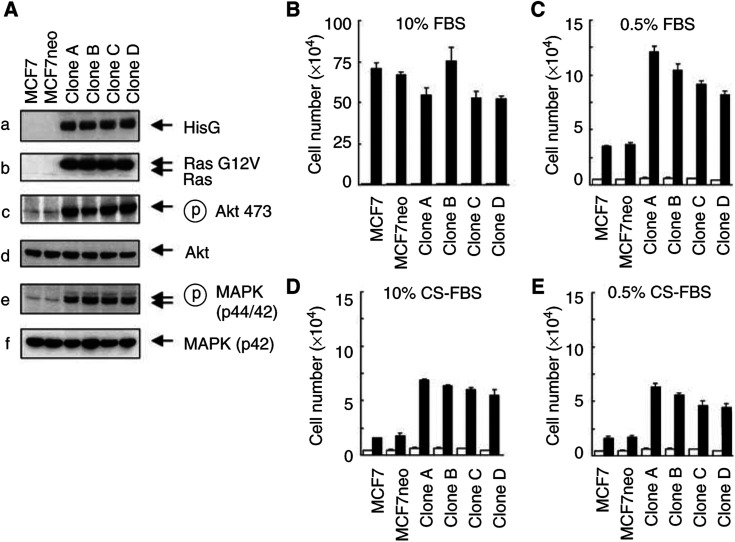
Establishment and characterisation of MCF7 human breast cancer cells expressing constitutively active RasG12V. (**A**) Parental MCF7 cells, control-vector-transfected MCF7 cells, and four selected MCF7 cell clones expressing RasG12V were cultured overnight in a medium containing 0.5% charcoal-stripped fetal bovine serum (CS-FBS). The cells were then harvested for Western blot analyses with various indicated antibodies. (**B–E**) The cells described in (**A**) were cultured for 5 days in a medium containing 10 or 0.5% fetal bovine serum (FBS) (B, C) or 10 or 0.5% CS FBS. Cells were counted with a Coulter counter.

We transfected MCF7 cells with a constitutively active Ras (RasG12V) expression vector and selected the tranfectants with 400 *μ*g ml^−1^ G418 for positive clones. Over 10 clones expressing various levels of RasG12V were obtained. Four representative clones with the highest levels of RasG12V expression are shown in Figure 1A

(gels a and b). Compared with parental or control-vector-transfected MCF7 cells, the MCF7RasG12V cells exhibited marked increases in the levels of phosphorylated Akt and MAPK (Figure 1A gels c and e), indicating that the transfected RasG12V was functionally constitutively active. The increases in the activation of Akt and MAPK were not accompanied by changes in their individual protein levels (Figure 1A gels d and f). While there was no significant difference in the proliferation rate between the RasG12V-transfected cells and control-vector-transfected cells growing in a regular culture medium (Figure 1B), expression of the RasG12V enhanced the survival of the cells cultured in a low-serum medium (Figure 1C) or in medium with CS serum (hormone-depleted) (Figure 1D and E).

### Resistance of MCF7RasG12V cells to doxorubicin, paclitaxel, and 5-fluorouracil (5-FU)

**Figure 2:**
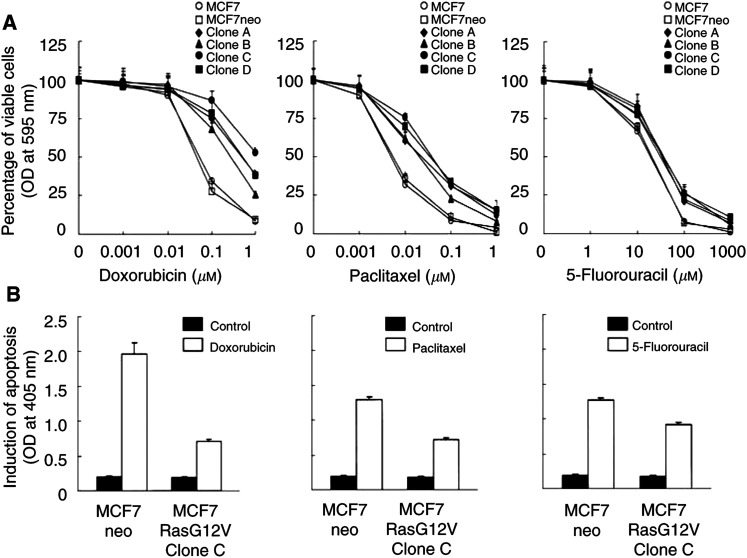
Increased resistance of MCF7RasG12V breast cancer cells to treatment with doxorubicin, paclitaxel, and 5-fluorouracil. (**A**) The cells described in Figure 1 were pulse-exposed to doxorubicin, paclitaxel, or 5-fluorouracil for 4 h at the indicated concentrations. The cells were then cultured for an additional 7 days with a drug-free medium supplemented with 10% CS-FBS medium. Cell viabilities after chemotherapy were determined by an MTT assay as described in Materials and Methods and were expressed as the percentage of the value of untreated cells. (**B**) One representative cell clone (clone C) was treated with doxorubicin, paclitaxel, or 5-fluorouracil for 8 h, followed by additional culture for 16 h with 10% CS-FBS medium. Cells were harvested and subjected to apoptosis ELISA.

Compared with control-vector-transfected MCF7 cells, MCF7RasG12V clones showed increased resistance to the treatment with doxorubicin, paclitaxel, or 5-fluorouracil (Figure 2A

). The IC_50_ of doxorubicin and paclitaxel increased approximately 10-fold, with a lesser increase in the IC_50_ of 5-fluorouracil. The increased resistance to each of the individual drugs was associated with reduced induction of apoptosis by the antineoplastic agents, assayed by an apoptosis ELISA (Figure 2B).

### Involvement of PI-3K and MEK/MAPK in Ras-mediated drug resistance

**Figure 3:**
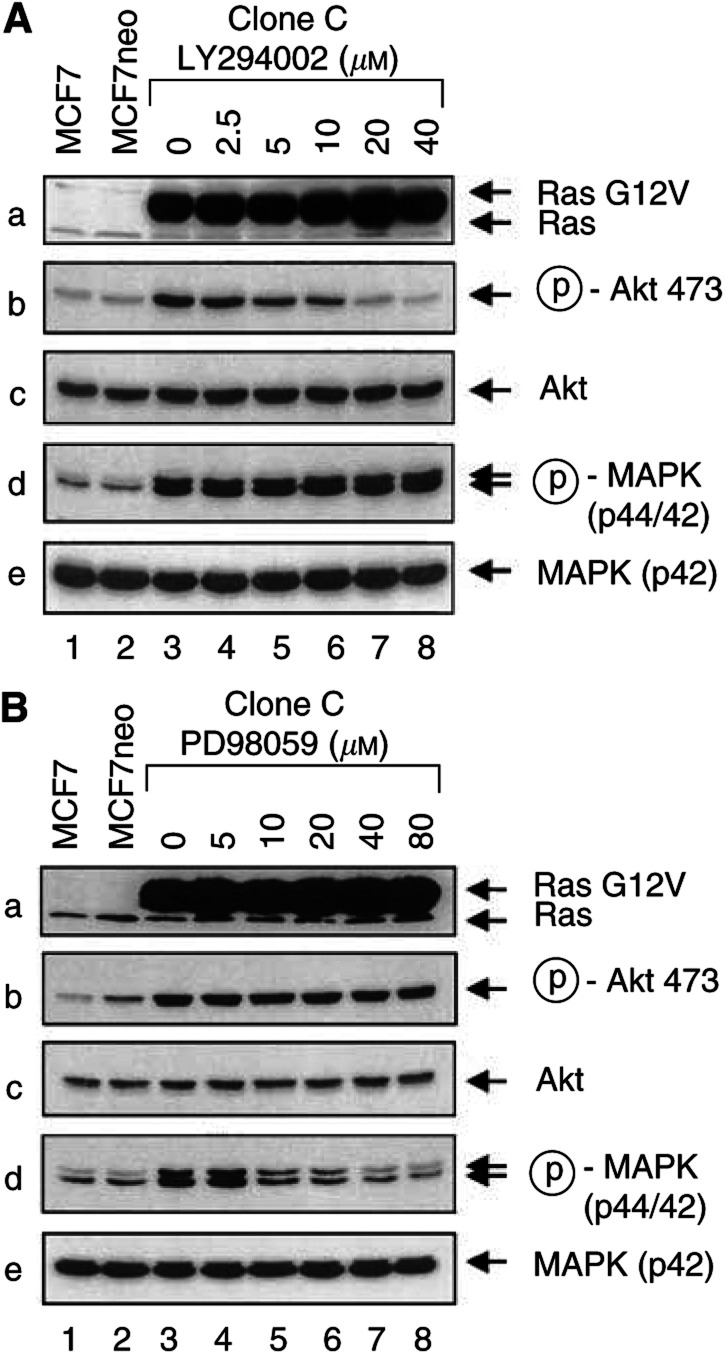
Noncrossing inhibition of RasG12V-induced activation of PI-3K and MEK by their respective specific inhibitors (PD98059 and LY294002). MCF7RasG12V clone C cells were exposed to increasing concentrations of PD98059 as indicated (**A**) or increasing concentrations of LY294002 as indicated (**B**) in a medium containing 0.5% CS FBS for 16 h, accompanied by parallel cultures of parental MCF7 cells and control-vector- transfected cells in 0.5% CS FBS medium without PD98059 or LY294002. Cells were collected for Western blot analysis for the levels of Ras (a), phosphorylated Akt (b), total Akt (c), phosphorylated MAPK (d), and MAPK (p42) (e).

**Figure 4:**
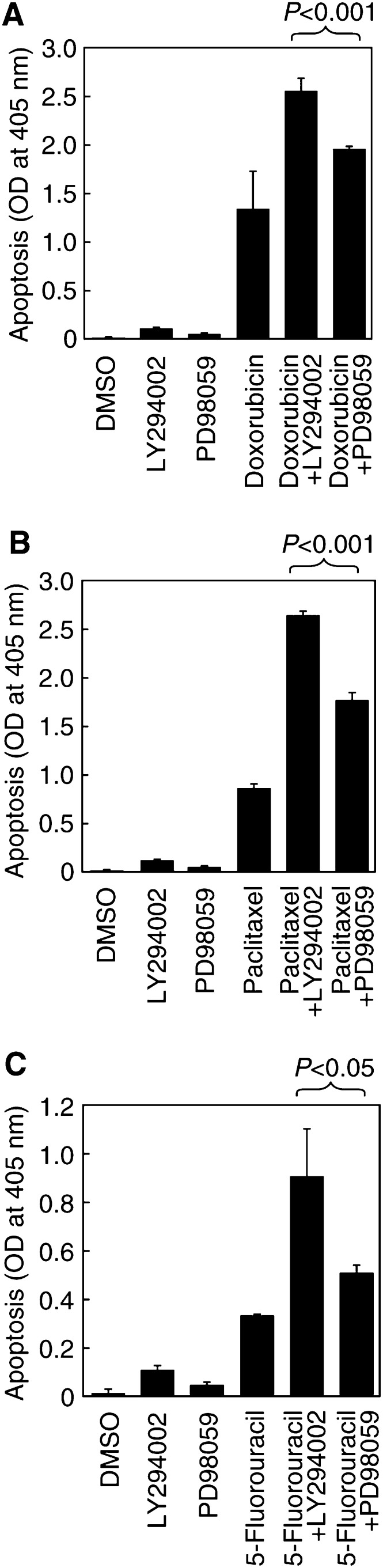
Enhancement of drug-induced apoptosis by the PI-3K-specific inhibitor LY294002 and the MEK-specific inhibitor PD98059 on MCF7RasG12V cells. MCF7RasG12V cells were cultured for a 16-h pre-drug period with or without 20 *μ*m LY294002 or 40 *μ*m PD98059, 8-h pulse exposure of the cells to 2.5 *μ*m doxorubicin (**A**), 5 *μ*m paclitaxel (**B**), or 40 mm 5-fluorouracil (**C**), and a 16-h postdrug period in 10% CS-FBS. Cells were then harvested for apoptosis ELISA. The statistical differences between LY294002 and PD98059 in enhancing the cytotoxicity of drugs (doxorubicin, paclitaxel, or 5-fluorouracil) were calculated by Student's *t*-test.

We then arbitrarily selected the MCF7RasG12V clone (clone C) and investigated the potential roles of the two major Ras downstream pathways, PI3K/Akt and MEK/MAPK, in Ras-mediated resistance of MCF7RasG12V cells to treatment with paclitaxel, doxorubicin, and 5-fluorouracil. We first examined whether inhibition of PI-3K with its specific inhibitor, LY294002, enhanced the apoptosis of the MCF7RasG12V cells following treatment with the chemotherapeutic agents. Within the dose range from 0 to 40 *μ*M LY294002, there was an LY294002-dose-dependent inhibition of PI-3K activity, as demonstrated by reduced phosphorylation of Akt by Western blot analysis with Akt serine-473 phosphorylation-specific antibodies. At the dose of 20 or 40 *μ*M, LY294002 nearly completely inhibited RasG12V-induced PI-3K/Akt activation (Figure 3A

, gels b, lanes 7 or 8 *vs* lanes 1 or 2). In contrast, within the given dose range of LY294002, there was no effect of LY294002 on the activity of the MEK/MAPK pathway; the Ras-induced increase in MEK/MAPK activity was unchanged (Figure 3A, gels d, lane 7 or 8 *vs* lanes 1 or 2), indicating the relative specificity of LY294002 in our experimental system in inhibiting the PI-3K/Akt pathway. We found that the treatment of MCF7RasG12V cells with LY294002 markedly sensitised the cells to doxorubicin-induced apoptosis (Figure 1A). Similar enhancement of sensitivity by LY294002 was found in MCF7RasG12V cells treated with paclitaxel or 5-fluorouracil (Figure 4B and C

).

We simultaneously examined whether inhibition of MEK with its specific inhibitor, PD98059, would enhance apoptosis of the MCF7RasG12V cells following treatment with the chemotherapeutic agents. Similar to the specificity of inhibition of LY294002 (within the given dose range) on PI-3K, there was a PD98059-dose-dependent inhibition of MEK activity, as demonstrated by reduced phosphorylation of MAPK by Western blot analysis with MAPK threonine-202 phosphorylation-specific antibodies. At doses between 40 and 80 *μ*M, PD98059 nearly completely inhibited RasG12V-induced MEK/MAPK activation (Figure 3B, gel d, lane 7 or 8 *vs* lanes 1 or 2), but had no effect on the activity of the PI-3K/Akt pathway. The Ras-induced increase in PI-3K/Akt activity remained, indicating the relative specificity of PD98059 in our experimental system in inhibiting MEK/MAPK pathways. Similar to the results with LY294002 treatment, inhibition of the MEK/MAPK pathway with PD98059 sensitised the MCF7RasG12V cells to treatments with doxorubicin, paclitaxel, and 5-fluorouracil, but it appears that, in all three cases, inhibition of PI-3K with LY294002 is statistically more effective than inhibition of MEK in enhancing drug-induced apoptosis (Figure 4).

**Figure 5:**
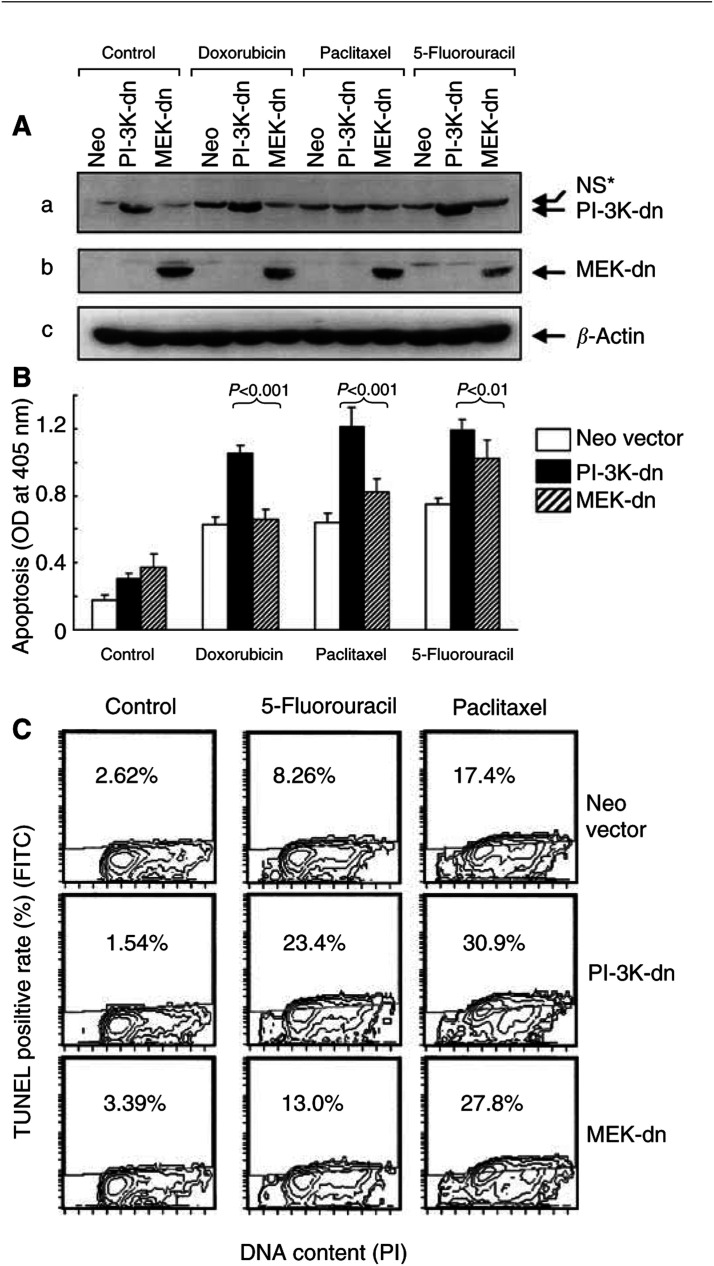
Effects of dn PI-3K (Δp85) and dn MEK (K97 M) on Ras-induced drug resistance. MCF7RasG12V cells were transfected with plasmid containing HA-tagged MEK (K97 M) or HA-tagged PI-3K (Δp85) for 16 h for transient expression, followed by a 8-h exposure to doxorubicin, paclitaxel, or 5-fluorauracil, and a 16-h postdrug period in a medium containing 10% CS FBS. The cells were then harvested for detection of the expressions of the dn forms of PI-3K (A, gel a) or MEK (A, gel b) by Western blot analyses with an anti-HA antibody, and for determination of apoptosis by ELISA (**B**) and by TUNEL (**C**). The statistical differences between the cells expressing dn PI-3K (Δp85) and dn MEK (K97 M) in enhancing the cytotoxicity of drugs (doxorubicin, paclitaxel, or 5-fluorouracil) were calculated by Student's *t*-test. dn: dominant negative; NS^*^=nonspecific signals.

Although both LY294002 and PD98059 showed relative specificity in inhibiting PI-3K and MEK, respectively, there were still possibilities that the two compounds may inhibit additional kinases other than PI-3K or MEK. To further confirm our results obtained with LY294002 and PD98059, we investigated whether expression of dominant-negative (dn) PI-3K (delta p85) or dn MEK (M97K) will have similar effects on sensitising the MCF7RasG12V cells to the drug-induced apoptosis. Figure 5A

shows the transient expressions of dn PI-3K and dn MEK in MCF7RasG12V cells, respectively. Compared with control-vector-transfected cells, expression of a dn PI-3K sensitized the MCF7RasG12V cells to doxorubicin-, paclitaxel-, and 5-fluorouracil-induced apoptosis, assayed by the apoptosis ELISA (Figure 5B). In contrast, expression of a dn MEK showed different effects: nearly no effect on doxorubicin-induced apoptosis, a slight effect on paclitaxel-induced apoptosis, and a more apparent effect on 5-fluorouracil-induced apoptosis. To provide an additional assay to measure the sensitisation of dn PI-3K and dn MEK on drug-induced apoptosis, we performed TUNEL assay (Figure 5C). Transient expression of either dn PI-3K or dn MEK showed similar sensitisation of paclitaxel- and 5-fluorouracil-induced apoptosis in MCF7RasG12V cells. We were unable to detect doxorubicin-induced apoptosis by TUNEL, possibly due to the interference of an inherent fluorescence of doxorubicin.

## DISCUSSION

The mechanism of drug resistance involves not only the delivery of drugs into targeted cancer cells but also the ability of cancer cells to survive under drug challenge, which is collectively contributed by multiple intrinsic and extrinsic factors. Recent interest and effort in reducing the resistance of cancer cells to chemotherapy have been focused on modulation at various levels of signalling pathways in cancer cells. In our current study, we demonstrated an unequal contribution of the two important Ras downstream pathways, PI-3K and MEK/MAPK, in mediating increased drug resistance in MCF7 human breast cancer cells following the expression of a constitutively active Ras (H-RasG12V). This experimental setting was undertaken to reflect not only the oncogenic Ras mutation *per se*, but in a boarder sense, the Ras protein activated by receptor tyrosine kinase-mediated signal transduction, which is commonly seen in breast cancer. We found that the resistance of MCF7 cells expressing H-RasG12V to the commonly used breast cancer chemotherapeutic agents (doxorubicin, paclitaxel, and 5-fluorouracil) was well correlated with apoptosis induced by the antineoplastic agents.

Cells often receive multiple stimuli. Given the complexity of Ras-mediated signal transduction, none of its downstream pathways function alone. In order to develop molecular-targeted approaches for sensitising cancer cells to chemotherapy, it is important to elucidate the downstream pathways that play major roles in Ras-mediated drug resistance of cancer cells to chemotherapy. The MEK/MAPK and PI-3K pathways are at the bifurcation point of Ras-mediated downstream pathways. Our experimental goal was to determine whether both MEK/MAPK and PI-3K pathways contribute to Ras-mediated drug resistance, and if so, which of the two pathways plays a major role in mediating Ras activation-induced drug resistance. By using both specific inhibitors and dominant negative expression vectors of MEK or PI-3K, we found that PI-3K seems to play a major role in drug resistance in our experimental setting, because inhibition of PI-3K by treatment of the cells with the PI-3K specific inhibitor LY294002 or by expression of a dominant negative PI-3K expression vector (Δp85) showed a statistically significant better sensitisation than inhibition of MEK with similar approaches in MCF7RasG12V cells following treatment with doxorubicin, paclitaxel, or 5-fluorouracil. An important caveat is that, in our experimental setting, we used the dose of LY294002 (20 *μ*M) that completely inhibited Ras-mediated PI-3K activation (demonstrated by reduced phosphorylation of Akt to the level comparable to that in control-vector-transfected cells, shown in Figure 3A, gel b), and that did not affect the activity of MEK (demonstrated by unchanged phosphorylation of MAPK, shown in Figure 3A, gels d). Similarly, we used the dose of PD98059 (40 *μ*M) that completely inhibited the activity of MEK without affecting the activity of PI-3K, as demonstrated by reduced phosphorylation of MAPK to the level comparable to that for control- vector-transfected cells and by an unchanged level of Akt phosphorylation (shown in Figure 3B gels b and d). This experimental condition allowed us to rule out any potential variation that may result from insufficient or cross inhibition of PI-3K or MEK by LY294002 or PD98059. Furthermore, because LY294002 or PD98059 may inhibit other kinases in addition to PI-3K or MEK, we used the dominant negative expression vectors of PI-3K and MEK, respectively, in our experiments and obtained comparable results.

Akt is one of the major effector molecules following PI-3K activation. Akt is activated by PDKs following its recruitment to the cell membrane by phosphatidylinositol 3,4,5-triphosphate (PtdIns-(3,4,5)-*P3*) and phosphatidylinositol 3,4-biphosphate (PtdIns-(3,4)-*P2*), which are generated by PI-3K. Recent evidence indicates that Akt is frequently activated in human breast cancer. The activated status of Akt may lead to breast cancer patients being more prone to relapse with distant metastasis (Perez-Tenorio and Stal, 2002). In breast cancer, the increase in Akt kinase activity may mainly result from the activities of Akt upstream regulatory signals produced by overexpression or activation of the EGF receptor family. Less commonly, it could also result from the mutational activation of Ras oncogene that activates Akt through the PI-3K pathway (Datta *et al*, 1996; Liu *et al*, 1998) or from the mutational inactivation of the PTEN tumour-suppressor gene that normally inhibits Akt activity by dephosphorylating the PI-3,4,5-*P_3_* and PI-3,4-*P2* produced by PI-3K (Stambolic *et al*, 1998; Ramaswamy *et al*, 1999). Although the mechanisms have not yet been fully characterised, activated Akt signalling is believed to promote cell survival by regulating several key molecules that are involved in apoptosis, such as Bad (Datta *et al*, 1997; del Peso *et al*, 1997), caspase-9 (Cardone *et al*, 1998), FKHRL1 (Brunet *et al*, 1999), CREB (Du and Montminy, 1998), and IKK*α* (Ozes *et al*, 1999).

Earlier observations reported that IGF-I can rescue MCF7 cells from doxorubicin-induced apoptosis in a PI-3K-dependent, but not MAPK-dependent manner (Gooch *et al*, 1999). In contrast, IGF-I rescued MCF7 cells from paclitaxel-induced apoptosis, which required both PI-3K and MAPK, suggesting that the drug mechanism-specific action in IGF-I attenuated the response of breast cancer cells to doxorubicin and paclitaxel (Gooch *et al*, 1999). In our experimental setting with the MCF7 cells expressing constitutively active Ras, we found that the apoptosis induced by paclitaxel or doxorubicin can be enhanced by inhibition of either PI-3K or MAPK, but inhibition of the PI-3K appears to be more effective, suggesting that both pathways are unequally involved in rescuing the cells from drug-induced apoptosis.

In summary, our results indicate that the PI-3K pathway may play a more important role in mediating receptor tyrosine kinase-mediated resistance to chemotherapy and suggest that PI-3K/Akt might be a critical target molecule for anticancer intervention in breast cancer.
